# Concentrated Ambient Particles Alter Myocardial Blood Flow during Acute Ischemia in Conscious Canines

**DOI:** 10.1289/ehp.11380

**Published:** 2008-09-10

**Authors:** Carlo R. Bartoli, Gregory A. Wellenius, Brent A. Coull, Ichiro Akiyama, Edgar A. Diaz, Joy Lawrence, Kazunori Okabe, Richard L. Verrier, John J. Godleski

**Affiliations:** 1 Molecular and Integrative Physiological Sciences Program, Department of Environmental Health, Harvard School of Public Health, Boston, Massachusetts, USA; 2 Department of Medicine, Beth Israel Deaconess Medical Center, Boston, Massachusetts, USA; 3 Department of Biostatistics, Harvard School of Public Health, Boston, Massachusetts, USA; 4 Sanyo National Hospital, Yamaguchi, Japan; 5 Department of Pathology, Brigham and Women’s Hospital, Boston, Massachusetts, USA

**Keywords:** coronary vasoconstriction, microspheres, myocardial blood flow, myocardial ischemia, particulate air pollution

## Abstract

**Background:**

Experimental and observational studies have demonstrated that short-term exposure to ambient particulate matter (PM) exacerbates myocardial ischemia.

**Objectives:**

We conducted this study to investigate the effects of concentrated ambient particles (CAPs) on myocardial blood flow during myocardial ischemia in chronically instrumented conscious canines.

**Methods:**

Eleven canines were instrumented with a balloon occluder around the left anterior descending coronary artery and catheters for determination of myocardial blood flow using fluorescent microspheres. Telemetric electrocardiographic and blood pressure monitoring was available for four of these animals. After recovery, we exposed animals by inhalation to 5 hr of either filtered air or CAPs (mean concentration ± SD, 349.0 ± 282.6 μg/m^3^) in a crossover protocol. We determined myocardial blood flow during a 5-min coronary artery occlusion immediately after each exposure. Data were analyzed using mixed models for repeated measures. The primary analysis was based on four canines that completed the protocol.

**Results:**

CAPs exposure decreased total myocardial blood flow during coronary artery occlusion by 0.12 mL/min/g (*p* < 0.001) and was accompanied by a 13% (*p* < 0.001) increase in coronary vascular resistance. Rate–pressure product, an index of myocardial oxygen demand, did not differ by exposure (*p* = 0.90). CAPs effects on myocardial blood flow were significantly more pronounced in myocardium within or near the ischemic zone versus more remote myocardium (*p* interaction < 0.001).

**Conclusions:**

These results suggest that PM exacerbates myocardial ischemia by increased coronary vascular resistance and decreased myocardial perfusion. Further studies are needed to elucidate the mechanism of these effects.

Transient elevations in ambient levels of particulate matter (PM) have been linked to an increased risk of acute cardiovascular events, including myocardial infarction, decompensation of heart failure patients, and stroke (Brook et al. 2004). Recent studies suggest that acute events that lead to hospitalization or death may represent only a small fraction of the acute cardiovascular effects of PM (Mittleman 2007). For example, studies in patients with implantable cardioverter defibrillators suggest an association between PM and the risk of life-threatening ventricular arrhythmias (Dockery et al. 2005; Rich et al. 2005). Similarly, although multiple studies have identified an association between short-term changes in PM and the risk of acute myocardial infarction (D’Ippoliti et al. 2003; Peters et al. 2001; Zanobetti and Schwartz 2005), more recent studies suggest a potential link between PM and unstable angina (Pope et al. 2006).

Several experimental (Godleski et al. 2000; Mills et al. 2007; Wellenius et al. 2003) and observational (Gold et al. 2005; Pekkanen et al. 2002) studies suggest that acute PM exposure may be associated with enhanced myocardial ischemia as determined by ST-segment changes on the electrocardiogram. Two such studies from our group found that conscious dogs exposed to concentrated ambient particles (CAPs) experienced greater ST-segment changes during transient coronary artery occlusion compared with dogs exposed to filtered air (Godleski et al. 2000; Wellenius et al. 2003). The above studies suggest that PM-related changes in myocardial blood flow may be responsible for the observed effects, but this hypothesis has not been directly examined. Our objective in this study was to test the hypothesis that acute exposure to CAPs reduces myocardial blood flow during transient myocardial ischemia. We tested this hypothesis in chronically instrumented conscious dogs exposed to either CAPs or filtered air, using fluorescent microspheres to directly measure myocardial blood flow.

## Materials and Methods

### Surgical preparation

Animals were treated humanely and with regard for alleviation of suffering using protocols approved by the Harvard Medical Area Standing Committee on Animals. We obtained adult female mixed-breed canines weighing 14–18 kg from Marshall Laboratories (North Rose, NY, USA) for use in this study. Anesthesia and instrumentation surgeries were performed as previously described (Bartoli et al. 2008b; Wellenius et al. 2003). Briefly, a left lateral thoracotomy was performed under inhalation anesthesia. A hydraulic balloon occluder was installed around the left anterior descending (LAD) coronary artery. Silicone catheters (7 French; Access Technologies, Skokie, IL, USA) were chronically implanted in the left atrium and descending aorta, tunneled from the thoracotomy incision to the back, and attached to a subcutaneous vascular access port (Polysulfone GPVu; Access Technologies). In four animals, a radiotelemetry unit (D70-CCTP; Data Sciences International, St. Paul, MN, USA) capable of measuring femoral arterial blood pressure and electrocardiogram was implanted in the left flank. In all animals, a permanent tracheostomy was surgically created to facilitate inhalation exposure (Bartoli et al. 2008a). After surgery, animals recovered for a minimum of 3 weeks before participating in experiments. Each dog was gradually acclimated to the laboratory and exposure conditions to minimize any stress associated with the experiments.

Eleven animals underwent surgery as described above. The hydraulic coronary occluder failed in three animals, and three additional animals developed a myocardial infarction; we excluded these six animals from analysis. The aortic catheter failed in one additional animal, which we excluded from the primary analysis (*n* = 4) but included in a sensitivity analysis (*n*= 5), as described below.

### Exposure technology and characterization

To evaluate the effects of ambient PM on myocardial blood flow, we repeatedly exposed pairs of animals via the permanent tracheostomy to 5 hr of either filtered air or CAPs. The characteristics of the Harvard Ambient Particle Concentrator (HAPC) have been previously described (Godleski et al. 2000; Sioutas et al. 1995). Briefly, the HAPC concentrates ambient particles ≤ 2.5 μm in aerodynamic diameter to approximately 30 times ambient levels without altering its size distribution or chemical composition. Particles with diameters > 2.5 μm are removed upstream of the HAPC, whereas ultrafine particles (≤ 0.1 μm in aerodynamic diameter) and ambient gases are neither enriched nor excluded.

Exposures typically took place between 0800 and 1430 hours each day. We meas ured CAPs mass concentration using a tapered-element oscillating microbalance (TEOM series 1400a; Rupprecht and Patashnick Co. Inc., East Greenbush, NY, USA); particle number concentration using a condensation particle counter (CPC model 3022A; TSI, Inc., Shoreview, MN, USA), and black carbon concentration by optical transmission (Aethalometer model AE-9; Magee Scientific, Berkeley, CA, USA), as previously described (Godleski et al. 2000).

### Experimental design

**Figure 1:**
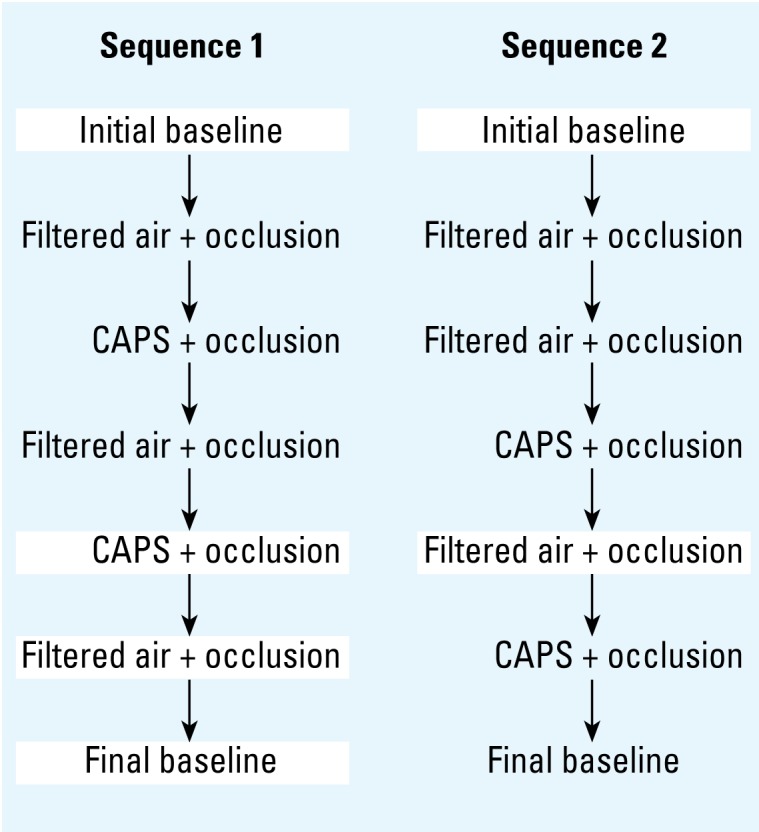
Exposure protocol for assessing the effects of CAPs on myocardial blood flow during coronary artery occlusion. After recovery from surgery, dogs were randomized to one of two exposure sequences. Myocardial blood flow was determined with fluorescent microspheres at baseline (no exposure, no coronary artery occlusion) and during a 5-min coronary artery occlusion immediately after 5 hr exposure to either CAPs or filtered air. Exposures in the same animal were separated by a minimum of 1 week.

To evaluate the effects of CAPs on myocardial blood flow, we repeatedly exposed pairs of animals to 5 hr of either filtered air or CAPs in a crossover protocol (Figure 1). Exposures in the same animal were separated by a minimum of 1 week. Immediately after each exposure, animals underwent two 5-min occlusions of the LAD coronary artery separated by a 20-min rest period. Acute myocardial ischemia during each occlusion was confirmed by observing real-time electrocardiographic changes. We evaluated myocardial blood flow with fluorescent microspheres during the second occlusion after 3 min of ischemia. This occlusion protocol is consistent with our prior work in this animal model showing that CAPs exacerbate myocardial ischemia during the second LAD occlusion (Godleski et al. 2000; Wellenius et al. 2003).

To control for changes in myocardial blood flow due to repeated microsphere injection, each animal received a baseline injection of microspheres (initial baseline) without intervention 1 week before the first CAPs or filtered air exposure. One week after the last CAPs or filtered air exposure, animals received a second baseline injection of microspheres (final baseline) without exposure or intervention.

### Measurement of myocardial blood flow

We measured myocardial blood flow using standard fluorescent microsphere techniques as previously described (Bartoli et al. 2008b). The principle of this technique is as follows. Fluorescent microspheres (15 μm diameter) were injected into the left atrium, where they were mixed with blood in the left ventricle, ejected into the aorta, and distributed throughout the body in a pattern similar to that of red blood cells. Microspheres of this diameter should lodge within the smallest precapillary arterioles based on regional tissue blood flow patterns. Postmortem analyses quantified relative differences in blood flow among organs or within regions of a single organ. We used blood sampled from the descending aorta at a known rate using a calibrated withdrawal pump (Harvard Apparatus; Holliston, MA, USA) for 100 sec to determine the absolute flow to tissue samples in milliliters per minute or milliliters per minute per gram of tissue. Microspheres with different fluorescent properties permit multiple determinations of myocardial blood flow over time in the same animal.

For each determination, microspheres were vortexed for 1 min and sonicated in an ultrasonic water bath for 15 min before injection into the left atrium via the vascular access port. The bolus of microspheres was injected over a period of 10 sec, followed by a flush of 5 mL warmed heparinized saline (38°C; 20 IU/mL). Blood was sampled from the descending aorta at a rate of 7.75 mL/min using a calibrated Harvard Apparatus withdrawal pump for 100 sec starting 10 sec before the microsphere injection.

**Figure 2:**
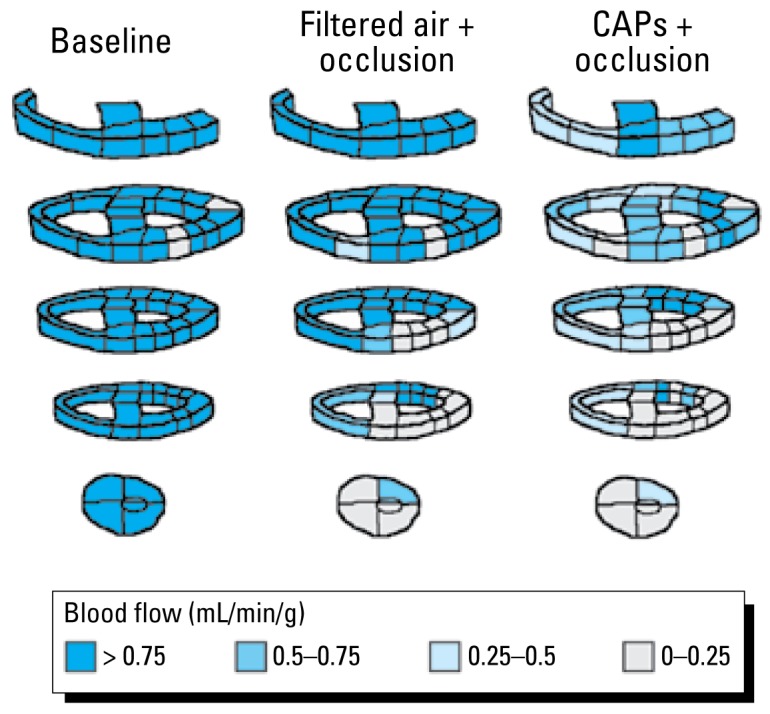
Transverse sections through the ventricles showing myocardial blood flow in one animal at baseline (left), during coronary artery occlusion immediately after 5 hr exposure to filtered air (center), and during coronary artery occlusion immediately after 5 hr exposure to CAPs (right). At baseline, myocardial blood flow is uniform except for a minor flow deficit at the location of hydraulic balloon occluder implantation. During coronary artery occlusion after exposure to filtered air, myocardial blood flow was diminished in the anterolateral left ventricular myocardium. During coronary artery occlusion after exposure to CAPs, myocardial blood flow to the same region was further diminished.

After euthanasia, the heart and surrounding structures were harvested. The ventricles were sectioned into 40–60 pieces of approximately 1 g each and mapped on a three-dimensional grid (Figure 2). We sent tissue and reference blood samples to IMT/Stason Laboratories (Irvine, CA, USA) for automated digestion of tissue samples and counting of fluorescent microspheres using flow cytometry. Instantaneous flow values were calculated for each piece of myocardial tissue as milliliters per minute per gram of tissue.

### Hemodynamic measurements

Continuous arterial blood pressure was monitored and recorded throughout exposures in three animals (DSI Dataquest ART 3.1; Data Sciences International). Data were exported and analyzed using custom-designed Matlab software (Mathworks, Inc., Natick, MA, USA) to obtain 5-min averages for each outcome. We derived systolic, diastolic, mean, and pulse arterial pressures as well as heart rate from the arterial blood pressure signal. We defined coronary vascular resistance as mean arterial pressure divided by total myocardial blood flow (Shen et al. 1994), and rate–pressure product as the product of heart rate and systolic blood pressure (Rooke and Feigl 1982).

### Statistical analysis

We calculated descriptive statistics for exposure measures (CAPs mass concentration, particle number concentration, black carbon concentration) and cardiovascular parameters (coronary artery resistance, arterial blood pressure, rate–pressure product) and used graphical methods to assess the distribution of each outcome. Coronary artery resistance was log-transformed in all analyses to improve the normality assumption.

We assessed the effect of CAPs exposure on myocardial blood flow using multilevel linear mixed models (Fitzmaurice et al. 2004) containing exposure type (CAPs, filtered air, initial baseline, final baseline) as fixed effects and tissue piece and dog as random effects. This modeling approach appropriately accounts for the correlation between multiple measurements on the same piece of tissue over time as well as for the correlation between measurements made in different tissue pieces within the same animal. We evaluated dose–response relationships by replacing the CAPs binary term with the 5-hr mean CAPs mass concentration, particle number concentration, or black carbon concentration.

To assess whether the CAPs effects on myocardial blood flow differed in ischemic versus nonischemic tissue, we dichotomized each piece according to its location. A tissue piece was estimated to be in or near the ischemic zone if the average blood flow during ischemia after filtered air exposure was less than under nonischemic baseline conditions and outside of the ischemic zone otherwise. We assessed differences in CAPs response in the ischemic versus nonischemic zone by incorporating fixed effects terms for zone (ischemic vs. non-ischemic) and zone-by-exposure interactions.

In sensitivity analyses, we considered alternative models that treated dogs as fixed effects rather than random effects and included a linear trend of time to account for the possible effects on myocardial blood flow of repeat ischemic events or accumulation of microspheres in myocardial capillary beds over time. The results from these sensitivity analyses were not materially different from those of the primary analyses.

As noted above, no reference blood sample could be collected from one animal in which the aortic catheter failed. Without the reference blood sample, it was still possible to evaluate the effects of exposure on total myocardial blood flow under the unverifiable assumption that a similar number of microspheres was used for each determination. To include the data from this additional animal, we repeated the primary analysis using the number of microspheres in each tissue piece as the outcome rather than absolute values of flow. Specifically, the number of microspheres in each tissue piece was modeled using a Poisson log-linear mixed model containing fixed effects for exposure and random effects for tissue piece and each dog.

Linear mixed model analyses were performed using PROC MIXED (version 9; SAS Institute Inc., Cary, NC, USA). We implemented Poisson log-linear mixed models in SAS using the GLIMMIX macro. Descriptive statistics were computed in SAS, and graphical inspection of all data was performed using R statistical software (R Development Core Team 2005). All statistical tests are two tailed, and *p*< 0.05 is considered statistically significant.

## Results

Figure 2 illustrates regional myocardial blood flow in one animal during different experimental conditions. In this example, myocardial blood flow during coronary artery occlusion appears to be reduced to a greater extent after 5 hr of CAPs exposure compared with filtered air exposure.

**Figure 3:**
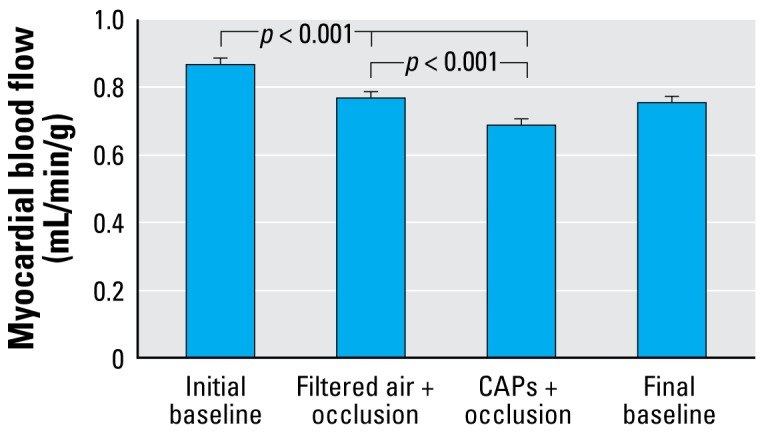
Total myocardial blood flow at baseline and during coronary artery occlusion after a 5 hr exposure to either filtered air or CAPs. Bars represent model-estimated mean ± SE from four dogs exposed to CAPs on 7 days and filtered air on 9 days.

Among four dogs that completed the exposure protocol, total myocardial blood flow during coronary artery occlusion was lower after CAPs exposure than after filtered air exposure (Figure 3). In mixed-effect models controlling for tissue piece and animal, CAPs exposure was associated with a statistically significant 0.12 mL/min/g (SE = 0.02; *p* < 0.001) reduction in total myocardial blood flow during coronary artery occlusion. In an analysis of the relative flow (rather than absolute flow; *n* = 5 dogs), CAPs exposure was associated with a 5.7% [95% confidence interval (CI), 1.7–9.5%; *p* = 0.005] reduction in myocardial blood flow versus filtered air.

We calculated coronary vascular resistance and rate–pressure product, a standard index of cardiac metabolic demand, during myocardial ischemia in three of the four animals from the primary analysis where data were available (Table 1). CAPs exposure was associated with a 13% (95% CI, 7.4–19.4%; *p* < 0.001) increase in coronary vascular resistance during coronary artery occlusion, whereas rate–pressure product remained unchanged (Table 1). Average values of systolic and diastolic blood pressure during LAD occlusion were higher after CAPs exposure versus filtered air exposure, but the difference was not statistically significant.

**Figure 4:**
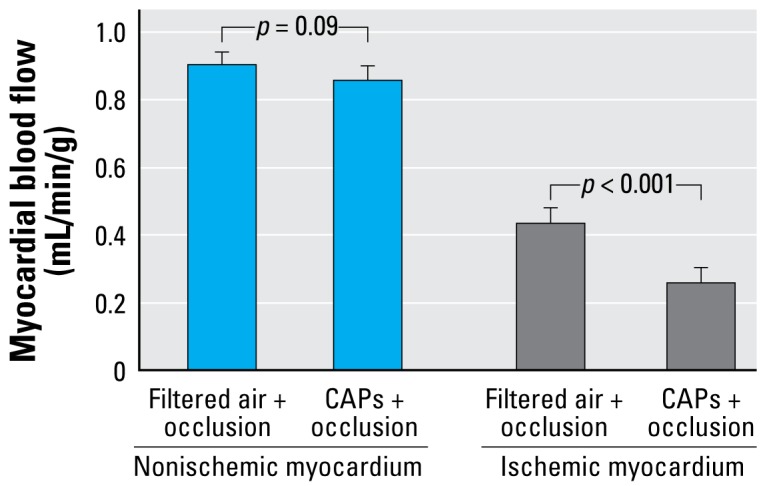
Myocardial blood flow in tissue not in the ischemic zone compared with tissue within or near the ischemic zone during coronary artery occlusion immediately after 5 hr exposure to either filtered air or CAPs. Bars represent model-estimated mean ± SE from four dogs exposed to CAPs on 7 days and filtered air on 9 days.

We assessed whether CAPs effects were similar in ischemic versus nonischemic myocardium (Figure 4). In tissue outside of the ischemic zone, CAPs exposure decreased myocardial blood flow during coronary artery occlusion by 0.05 mL/min/g (SE = 0.03; *p* = 0.09). In contrast, in tissue within or near the ischemic zone, CAPs exposure decreased myocardial blood flow during coronary artery occlusion by 0.22 mL/min/g (SE = 0.03; *p* < 0.001). The interaction term between CAPs exposure and zone was statistically significant (*p* < 0.001). The CAPs-induced increase in coronary vascular resistance was similarly greater within the ischemic zone as compared with tissue outside the ischemic zone (33.7 vs. 5.8%, respectively; *p* for interaction < 0.001).

### CAPs characteristics

Daily CAPs mass concentration ranged from 94.1 to 1556.8 μg/m^3^ (mean ± SD, 349.0 ± 282.6 μg/m^3^), particle number concentrations ranged from 3,000 to 69,300 particles/cm^3^ (20,381 ± 13,075 particles/cm^3^), and black carbon concentrations ranged from 1.3 to 32.0 μg/m^3^ (7.5 ± 5.6 μg/m^3^). Increases in CAPs mass, particle number, and black carbon concentrations were significantly associated with decreases in myocardial blood flow and increases in coronary vascular resistance during coronary artery occlusion (Table 2). We observed the strongest associations with particle number concentration.

## Discussion

The goal of this study was to evaluate the effects of short-term exposure to CAPs on myocardial blood flow. Our results show that acute exposure to CAPs decreases myocardial blood flow during subsequent coronary artery occlusion in chronically instrumented dogs. The decrease in myocardial blood flow was most pronounced in tissue within or near the ischemic zone and was accompanied by increases in coronary vascular resistance. Altered myocardial metabolic demand did not account for changes in myocardial blood flow.

Myocardial ischemia results from an imbalance between myocardial oxygen supply and demand. Several lines of evidence suggest that short-term exposure to ambient PM may lead to such an imbalance even in the absence of an acute myocardial infarction. We have previously shown in this animal model that occlusion-induced ST-segment changes (an electrocardiographic marker of myocardial ischemia) are more pronounced after CAPs exposure than after filtered air exposure (Godleski et al. 2000; Wellenius et al. 2003). Pekkanen et al. (2002) found an association between ambient PM levels 2 days earlier and risk of ST-segment depression during repeated submaximal exercise tests in a panel of subjects with stable coronary heart disease. Gold et al. (2005) found an association between ambient levels of black carbon, a marker of traffic-related PM, and ST-segment depression during rest and after brief periods of exercise. Mills et al. (2007) recently showed that a 1-hr controlled exposure to dilute diesel exhaust versus filtered air acutely increased exercise-induced ST-segment depression in males with stable coronary heart disease.

The results of the present study extend this existing literature by providing direct evidence that the exacerbation of myocardial ischemia observed in previous studies is due at least in part to impaired myocardial blood flow. Our observation that CAPs exposure did not alter cardiac metabolic demand as measured by the rate–pressure product is in agreement with the findings of Mills et al. (2007), as well as our previous study (Wellenius et al. 2003), and suggests that reduced myocardial blood flow rather than altered myocardial energetics may be the primary mechanism of PM-associated exacerbation of myocardial ischemia.

Our results do not directly identify the pathophysiologic mechanisms responsible for the observed effects of CAPs on myocardial blood flow, but may provide some insights. Our observation that CAPs exposure increases coronary vascular resistance during ischemia suggests an important role for PM-induced coronary vasoconstriction. Such a role for PM would be consistent with the findings by Brook et al. (2002) of decreased brachial artery diameter (but not endothelium-dependent vascular reactivity) after a 2-hr exposure to CAPs and ozone in healthy volunteers. PM exposure might instead affect coronary vascular reactivity, as suggested by findings of increased contractile response to endothelin-1 and decreased dilatory response to the nitric oxide donor sodium nitroprusside in the coronary arteries of apolipoprotein E knockout (*ApoE*^−/−^) mice exposed to diesel exhaust (Campen et al. 2005). In the setting of complete coronary artery occlusion, oxygen supply to the region at risk of ischemia depends primarily on the extent of recruitment of collateral vessels in the hypoperfused region. Conditions that reduce endothelium-derived nitric oxide have been shown to limit the dilator reserve of collateral vessels (Klassen et al. 1999; Traverse et al. 1998). Thus, it is plausible that PM-induced alterations in coronary collateral vasoactive responses could exacerbate myocardial ischemia by impairing collateral vessel recruitment in the setting of ischemia. Although our study did not test this hypothesis, the observation that the effects of CAPs on myocardial blood flow were more pronounced in tissue within or near the ischemic zone compared with more remote myocardium is consistent with this hypothesis.

In these experi ments, we assessed myocardial blood flow during the second of two sequential coronary artery occlusions 20 min apart. The first coronary artery occlusion each day represents ischemia-reperfusion injury that is expected to result in the initiation of a series of short-term adaptive responses collectively referred to as preconditioning (Vinten-Johansen et al. 2007). Thus, an alternative explanation for the results observed in the present study is that exposure to CAPs limited or delayed the protective effects of ischemia-reperfusion injury, which includes recruitment of coronary collateral vessels (Billinger et al. 1999).

The results of the present study do not directly implicate the PM sources or chemical constituents of ambient PM responsible for the observed effects. We found statistically significant inverse associations between myocardial blood flow and concentrations of CAPs mass, particle number, and black carbon, with the strongest association observed with particle number concentration. Given the location of the HAPC adjacent to a busy urban roadway, this finding may implicate traffic-related pollution as an important source. This notion is consistent with the study of Gold et al. (2005), which found that ambient black carbon, but not fine PM, was associated with enhanced ST-segment changes in a panel of Boston-area elderly subjects. Similarly, the Exposure and Risk Assessment for Fine and Ultrafine Particles in Ambient Air (ULTRA) study by Pekkanen et al. (2002) found that exercise-induced ST-segment changes in a Helsinki, Finland, population were associated with ultrafine PM, carbon monoxide, and nitrogen dioxide (in addition to fine PM), again suggesting a role for traffic-related pollution. Because of the relatively small sample size, we could not explore this hypothesis further in the context of the present study.

### Strengths and limitations

An important limitation of this study is the small number of animals used in the primary analysis. The surgical preparation and maintenance of the animals used in this study were technically challenging and costly. The unexpected high failure rate of animals prevented us from completing these experiments in a larger cohort as originally planned. When relatively few animals are studied, there is a risk that the observed effects could be driven by chance findings in just one animal. Our secondary analysis using the end point of number of spheres rather than flow allowed us to evaluate the effects of CAPs on myocardial blood flow in five animals. Encouragingly, the results of this analysis were qualitatively similar to those of the primary analysis based on four animals. Still, because of the relatively small sample size, we may have failed to detect important effects (large type 2 error).

This study has other potential limitations. First, dogs were exposed to CAPs via a permanent tracheostomy, which bypasses the nasopharynx. Irritant receptors located in the nostrils, nasopharynx, and upper trachea may participate in respiratory and cardiovascular responses (Coleridge and Coleridge 1984). Bypassing upper-airway receptors implicated in eliciting physiologic responses may exclude important pathophysiologic pathways, especially in dogs, which rely heavily on olfactory senses. Second, although dogs are an excellent cardiovascular model species for humans, species differences demand caution in extrapolation of results to human populations. The generalizability of these findings may be further limited because we studied only female dogs.

Notwithstanding these limitations, the present study contains several strengths, including the use of a large animal model of myocardial ischemia, direct measurement of myocardial blood flow, and exposure to real-world ambient PM at realistic concentrations. Additional studies are required to elucidate the mechanisms of the observed effects and to identify the sources or chemical constituents of PM responsible.

## Figures and Tables

**Table 1:** Average hemodynamic measures during a 5-min coronary artery occlusion after a 5-hr exposure to either CAPs or filtered air.

Measure	Filtered air	CAPs	*p*-Value^a^
Systolic blood pressure (mmHg)	114.2 ± 20.7	122.7 ± 15.0	0.20
Diastolic blood pressure (mmHg)	73.6 ± 9.1	79.5 ± 7.1	0.11
Mean arterial pressure (mmHg)	88.3 ± 12.0	93.7 ± 7.0	0.21
Pulse pressure (mmHg)	40.7 ± 14.7	43.0 ± 16.0	0.78
Heart rate (bpm)	109.4 ± 23.4	99.4 ± 20.2	0.19
Rate–pressure product (bpm × mmHg)	12,197 ± 1,984	12,031 ± 1,951	0.90

a *p*-Value is for test of mean response in CAPs-exposed versus filtered-air–exposed animals, which is estimated from a mixed-effects model with fixed effects for CAPs and random dog effects.

**Table 2:** Change in myocardial blood flow and coronary vascular resistance associated with an interquartile range increase in concentrations of CAPs mass, particle number, and black carbon.

	Myocardial blood flow			Coronary vascular resistance		
CAP metric	Effect^a^	95% CI	*p*-Value	Effect^b^	95% CI	*p*-Value
Mass	−0.07	−0.09 to −0.05	< 0.001	10.5%	8.7 to 12.4%	< 0.001
Particle number	−0.34	−0.38 to −0.30	< 0.001	17.3%	12.9 to 22.0%	< 0.001
Black carbon	−0.11	−0.15 to −0.07	< 0.001	11.9%	9.4 to 14.5%	< 0.001

a Represents change in myocardial blood flow associated with an interquartile range increase in each CAPs metric compared with filtered air.

b Represents percent increase in coronary vascular resistance associated with an interquartile range increase in each CAPs metric compared with filtered air. Interquartile ranges were 231.6 μg/m^3^ for mass concentration, 19,650 particles/cm^3^ for particle number concentration, and 6.5 μg/m^3^ for black carbon concentration.
